# A Novel Magnetic Bead-Based Differential DNA Extraction Method with Potential for High-Throughput Automation in Forensic Casework: A Proof-of-Concept Study

**DOI:** 10.3390/genes17070824

**Published:** 2026-07-19

**Authors:** Amy-Leigh Whittaker, William P. Allan, Mark W. Perlin, Laura J. Heathfield

**Affiliations:** 1 Division of Forensic Medicine and Toxicology, Department of Pathology, Faculty of Health Sciences, University of Cape Town, Observatory, Cape Town 7925, South Africa; 2 Cybergenetics, Pittsburgh, PA 15213, USA

**Keywords:** sexual offences, differential extraction, Macherey-Nagel, Cytiva, mixture resolution, information-preserving genotyping, TrueAllele^®^

## Abstract

**Background/Objectives**: Sexual offences remain a global challenge, disproportionality affecting developing and conflict-stricken countries. Differential DNA extraction (DDE) is standardly applied to intimate swabs collected in these cases to separate and purify sperm and epithelial fractions ahead of DNA profiling. Whilst DNA purification steps using magnetic bead technology are routinely automated in general forensic workflows, the separation step within DDE workflows usually relies on centrifugation, which is challenging to automate in a high-throughput manner. This proof-of-concept study aimed to develop a novel method to separate sperm and epithelial fractions using magnetic bead technology to enable a fully automated and high-throughput DDE workflow. **Methods**: First, the Macherey-Nagel NucleoMag^®^ Forensic DNA kit’s protocol for forensic samples was modified to be DDE-based, and different magnetic beads for the separation and purification steps were assessed on mock sexual offence samples. Once a working protocol was established, variables within the protocol were systematically adjusted to improve quality metrics and DNA profiling outcomes. The top-performing method was then assessed with decreased input biological material and on a post-coital swab. **Results**: The DDE protocol developed in this study identified Cytiva SeraSil-Mag^TM^ magnetic beads to successfully separate epithelial and sperm fractions, which will enable a fully automated and high-throughput DDE workflow for the first time. DNA extracted from the sperm fraction of mock sexual offence samples prepared with 1 μL of semen input yielded a mean Y-target DNA yield of 25.04 ng (SD = 15.11 ng), a median M:F ratio of 1:3.53 (range = 1:1.08–1:22.46) and a mean log(LR) of 12.01 (SD = 5.41) when the female contributor’s DNA profile was unknown. **Conclusions**: The proof-of-concept of a magnetic bead-based DDE method was successfully demonstrated across a range of different semen input volumes, and the benefit of information-preserving genotyping using the TrueAllele^®^ system was demonstrated.

## 1. Introduction

The prevalence of sexual offences is a global issue, but is particularly high in Africa, with national rates far exceeding global averages. The United Nations Children’s Fund (UNICEF) estimates that within sub-Saharan Africa, more than 79 million females have been subjected to a sexual offence before turning 18 years old [1]. Sexual offences have also been reported as a weapon of war in countries such as the Democratic Republic of Congo, where conflict-related sexual violence is rife [2,3].

Despite significant underreporting, a staggering 53,498 sexual offences were reported in South Africa in 2022/2023 [4], prompting the South African government to declare violence against women as a national disaster in 2025 [5]. With forensic DNA backlogs common in many state forensic laboratories, justice is untimely or non-existent for women who have experienced sexual violence [6,7,8,9]. The lengthy differential DNA extraction (DDE) process for post-coital swabs, combined with manually interpreting mixed DNA profiles, adds to the challenges that many laboratories experience with processing DNA evidence from sexual offences.

In sexual offence cases involving a female victim/survivor and male perpetrator(s), an intimate swab is collected from the victim/survivor as DNA evidence and typically contains DNA from all contributors. To identify the male perpetrator(s), the DNA mixture is separated, as completely as possible, into sperm (male) and epithelial (female) fractions for purification prior to downstream analyses. This can be achieved using DDE [10,11], which takes advantage of the presence of disulfide bonds within the sperm cell membrane. During the initial incubation, epithelial cells can be selectively lysed and removed while sperm cells remain intact. The sperm cells are subsequently lysed through the addition of dithiothreitol (DTT) [11].

The manual performance of DDE can be laborious, time-consuming and skill-dependent. In many forensic laboratories, DDE is performed in a semi-automated manner, whereby the separation of male and female DNA is achieved by centrifugation (not using an automated liquid handler), and the purification of the resulting fractions uses magnetic bead technology, which can be automated on an automated platform. Examples include the Sep&Prep Kit on the EZ2 Connect (QIAGEN, Hilden, Germany), the PrepFiler^TM^ Automated Forensic DNA Extraction Kit on the AutoMate Express Forensic DNA Extraction System (Applied Biosystems, Foster City, CA, USA) or the Maxwell^®^ DE System (Promega, Fitchburg, WI, USA) [12,13,14]. Although effective at separation, centrifugation is not easily automated, especially in a high-throughput manner. Indeed, a fully automated method using centrifugation, with limited throughput for 24 samples, has been described using the Hamilton AutoLys STAR system for separation, coupled with the HID EVOlution^TM^ system for purification [15]. Centrifugation, however, was achieved using proprietary components (AutoLys-A tubes), which is a limiting factor for resource-constrained laboratories in countries where sexual violence is high.

Despite a myriad of technologies being explored for the separation step within DDE workflows, the use of solely magnetic beads remains understudied. Magnetic beads are readily accessible, and their use is well-established in many molecular biology and forensic-based workflows, particularly for DNA purification [16]. Further, using magnetic beads eliminates the need for centrifugation and allows for scalable automation. To date, their use for separation of epithelial and sperm fractions within a DDE workflow has not been extensively investigated [17].

Another obstacle to the delivery of timely justice and the identification of male perpetrators is manually resolving mixed DNA profiles, as well as the reliance on interpretation thresholds. Often, male DNA is already present in substantially lower quantities than female DNA at sample collection. Male DNA loss can also occur during DDE, resulting in small amounts of male DNA in the sperm fraction. Consequently, some laboratories may choose to exclude this sample entirely from further downstream analyses as they fail to meet the pre-determined DNA concentration and/or male-to-female (M:F) ratio thresholds. Moreover, should samples advance to short tandem repeat (STR) profiling, they may yield ‘partial profiles’, possibly attributed to male allele peaks falling below the laboratory-established analytical threshold (AT), thereby complicating mixture resolution and producing an inconclusive result. This systematic loss of information at each step of the forensic DNA analysis workflow decreases the chances of justice being served. Moreover, this loss is unnecessary with information-preserving probabilistic genotyping systems such as TrueAllele^®^.

TrueAllele^®^ technology is a fully Bayesian STR genotyping system used to solve complex low-level DNA mixtures [18]. Its hierarchical probability model represents the STR data generation process as nested random variables. By modelling PCR amplification and baseline noise, no AT is needed and all data peaks can be considered. When given the DNA evidence data, the system infers hundreds of process parameters with probability. Multi-locus contributor genotypes are separated by marginalisation of the Markov Chain Monte Carlo (MCMC)-computed joint posterior probability distribution.

Several empirical validation studies have shown that the system accurately preserves DNA identification information [18,19,20,21]. TrueAllele^®^ contributor information is expressed as a likelihood ratio logarithm (log(LR)); therefore, the information is linearly related to the logarithmic amount of that contributor’s DNA [22]. Thus, the system is a useful computer tool for measuring the information that DNA data contains, and scientists regularly use it for this purpose [23,24].

Qualitative mixture interpretation methods, i.e., those which use peak height thresholds, are not well-suited to quantifying DNA evidence information, as they discard considerable STR data [18,19,25]. Counting detected alleles that pass an arbitrary threshold does not precisely measure all the available DNA information. Furthermore, commonly used DNA mixture statistics, like the combined probability of inclusion (CPI), essentially count the number of subjectively matched STR loci [26].

Overall, the continued sexual violence experienced in many countries, coupled with the ever-increasing forensic caseload, highlights the need for better DNA analysis workflows. A DDE method that is fully automatable for a high number of samples would be the first step in improving DNA processing rates. Since the purification component of the workflow is already high-throughput and automated, this research aimed to explore magnetic bead-based technology for the separation of epithelial and sperm DNA. This proof-of-concept study thus showcases the development of a novel, easily accessible DDE method that has the potential for high-throughput once automated. Secondly, it used the TrueAllele^®^ system in two ways: (1) to measure the new method’s extracted DNA information, and (2) to demonstrate the feasibility of a more informative casework process [18].

## 2. Materials and Methods

### 2.1. Method Overview

This study was conducted in three phases. Given the nature of this study, an overview of the phases will be described in this section, and the details will be provided in the sections that follow. Further, some results may be alluded to in the Materials and Methods, as results from one phase informed the methods of the next phase. The first phase focused on establishing the method and identifying the combination of magnetic beads to be selected for separation and purification. Five experiments were conducted, namely a baseline method and four different magnetic bead combinations (outlined in Section 2.5.1), and each experiment included eight mock sexual offence samples (*n* = (8 samples × 5 experiments) = 40 samples). The resultant DNA from these experiments was assessed using quantitative real-time PCR (qPCR), and the concentration of male DNA and associated male:female (M:F) ratios of the resulting sperm and epithelial fractions were recorded. The top-performing method was selected and applied to a larger sample size to verify results. In this phase, 20 μL of neat semen was used to create mock sexual offence samples, as the objective was to determine which beads performed best, without the limitation of low male DNA input.

Once the magnetic bead selection had been made, the second phase focused on increasing male DNA and decreasing female DNA in the resultant sperm fraction. In this phase, 24 method adjustments were made and systematically assessed through DNA quantification and DNA profiling. The volume of semen used to create mock sexual offence samples was initially kept at 20 μL (method adjustments 1–5) and was then reduced to 5 μL (method adjustments 4 (B), 6–22). For each method adjustment, six mock sexual offence samples were used (*n* = (6 samples × 24 method adjustments) = 144 samples), and the resultant sperm fraction Y-target DNA yield, M:F ratio, presence of male alleles within the DNA profile, and log(LR) were recorded. The top three performing methods were applied to an expanded sample size (*n* = (3 samples × 3 methods) = 9 samples).

The third phase evaluated the top three performing methods using nine mock sexual offence samples created using reduced biological material from both females and males (1 μL semen) (*n* = (9 samples × 3 methods) = 27 samples). After comparison, a single method was selected for application on an authentic post-coital swab. Again, DNA quality metrics and DNA profiling outcomes were recorded.

### 2.2. Participants and Sample Collection

Biological samples were collected from three female and three male participants. All participants were at least 18 years old, and all samples were self-collected by the participants.

From each female participant, 120 buccal swabs were collected over time (*n* = 120 buccal swabs × 3 participants = 360 buccal swabs) using polyester swabs (Copan, Brescia, Italy). In phases 1 and 2, buccal swabs were collected by rotating the swab on the inner cheek of the participant’s mouth for 15 s. In phase 3, when reduced biological material was tested, buccal swabs were stroked on the inner cheek of the participant’s mouth in an “up-down” motion five times (equivalent to 4 s). Importantly, to mimic a realistic forensic scenario, the decision was made not to perform cell counts or determine the concentration of DNA present within the semen and buccal swabs prior to mock sexual offence sample preparation.

Buccal swabs were selected to represent the female contributor, as their use has been previously documented in other developmental studies [27,28,29,30,31,32], and they are relevant to cases of oral sexual violence. Although the microbial and cellular environments of buccal and vaginal swabs differ, the primary source of DNA within both is epithelial cells. Furthermore, buccal swab collection is less invasive, making it suitable for large-scale sample collection. Therefore, buccal swabs were considered ethically appropriate for primary optimisation; whilst acknowledging that vaginal swabs would be the necessary sample to include in further optimisation experiments once a proof-of-concept has been demonstrated.

For each male participant, four semen samples were collected, each in a separate 50 mL centrifuge tube (NEST Biotechnology, Wuxi, China) (*n* = 4 semen samples × 3 participants = 12 semen samples), and a reference buccal swab was collected using a polyester swab (Copan, Brescia, Italy) (*n* = 1 buccal swab × 3 participants = 3 buccal swabs). Male participants were excluded if they had previously undergone a vasectomy or had knowledge of being azoospermic or oligospermic.

A post-coital swab, as well as reference buccal swabs, were also collected from a consenting couple six hours after consensual sexual intercourse and used for method comparison.

Buccal swabs and the post-coital swab were stored at −20 °C immediately after collection, while semen samples were stored at 4 °C until processing.

**Figure 1:**
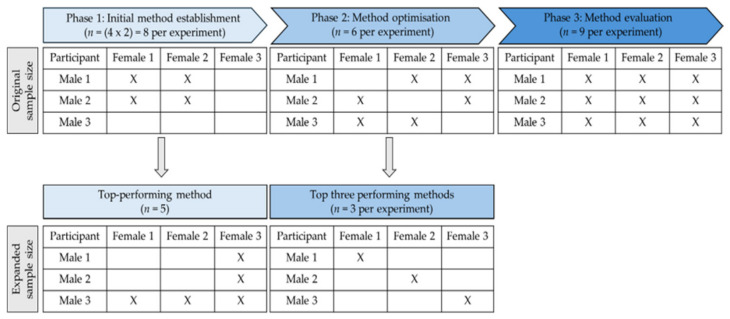
A breakdown of the combination of male and female participants (indicated by “X”) used to create mock sexual offence samples and the associated sample size per phase (Phase 1: *n* = 8 samples per experiment; 5 additional samples for the top-performing method, Phase 2: *n* = 6 samples per experiment; 3 additional samples per experiment for the top three performing methods, Phase 3: *n* = 9 samples per experiment).

Figure 1 illustrates the sample breakdown per phase and male-female combinations used to create the mock sexual offence samples.

### 2.3. Reference Sample DNA Extraction

One buccal swab per participant was used to generate a reference DNA profile. DNA was extracted from the reference buccal swabs using the QIAamp^®^ Investigator DNA Kit (QIAGEN, Hilden, Germany) according to the manufacturer’s protocol [33] and eluted in 50 μL elution buffer. These samples were quantified (Section 2.6) and profiled (Section 2.7) to identify each contributor within mixed resultant sperm fraction samples.

### 2.4. Baseline DDE Method Extraction

To establish ‘baseline’ results for DDE on mock sexual offence samples, the standard method used in the Biomedical Forensic Science Laboratory at the University of Cape Town (UCT) was first carried out. This entailed eight mock sexual offence samples being extracted using the QIAamp^®^ DNA Investigator Kit, according to the Isolation of Total DNA from Sexual Assault Specimens protocol within the manufacturer’s handbook [33]. An elution volume of 30 μL was used. These samples were quantified (Section 2.6) and used for comparative analysis during Phase 1 (Section 2.9).

### 2.5. DDE Method Development and Optimisation Using Mock Sexual Offence Samples

#### 2.5.1. Phase 1: Establishment of the Initial DDE Method

The objective of the first phase was to establish an initial DDE method, and this required the identification of suitable magnetic beads and the selection of the sperm cell lysis-reducing agent.

**Figure 2:**
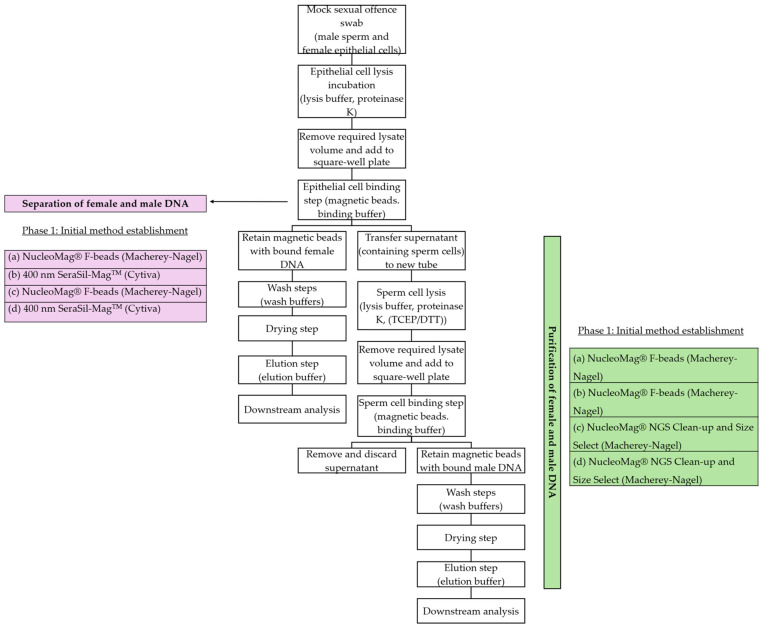
DDE method overview, using the NucleoMag^®^ DNA Forensic kit (Macherey-Nagel, Düren, Germany), according to the manual (excluding the use of TCEP), coupled with different magnetic bead separation and purification combinations (a–d) evaluated in Phase 1.

The manufacturer’s protocol for “Genomic DNA from forensic samples” using the NucleoMag^®^ Forensic DNA kit (Macherey-Nagel, Düren, Germany) [34] was modified to create a DDE method (Figure 2). This was done by performing an initial lysis step, with the exclusion of tris(2-carboxyethyl)phosphine (TCEP) (noted as “Epithelial cell lysis incubation” in Figure 2). The resultant lysate, containing both male and female DNA, was added to a square-well plate, and magnetic beads were added. The DNA bound to the magnetic beads was considered the epithelial fraction, while the removed supernatant (sperm fraction) underwent an additional lysis step with a sperm cell lysis reducing agent (DTT or TCEP). Thereafter, magnetic beads were added to the sperm fraction, and each fraction underwent standard purification. The wash steps for both fractions were performed according to the manufacturer’s protocol, and DNA was eluted to 30 μL with elution buffer FOE.

The separation and purification capabilities of three different magnetic beads were evaluated within the above protocol. The magnetic beads investigated included (i) NucleoMag^®^ F-beads (provided in the NucleoMag^®^ Forensic DNA kit), (ii) 400 nm SeraSil-Mag^TM^ (Cytiva, Marlborough, MA, USA) and (iii) NucleoMag^®^ NGS Clean-up and Size Select beads (Macherey-Nagel, Düren, Germany). These magnetic beads were paired in four different combinations (Figure 2), and a total of eight mock sexual offence samples per combination were used for each assessment (*n* = (8 samples × 4 combinations) = 32 samples).

The sperm cell lysis capabilities of DTT (Carl ROTH, Karlsruhe, Germany) and TCEP (included within the NucleoMag^®^ Forensic DNA kit) were simultaneously investigated in this phase. Therefore, once separated, the sperm fraction of half of these samples (*n* = (4 samples × 4 combinations) = 16 samples) underwent sperm cell lysis using DTT, while the other half underwent sperm cell lysis using TCEP. A DNA extraction blank was processed for each magnetic bead combination investigated. Following DDE, extracted DNA underwent DNA quantification as per Section 2.6. Resultant quantification data were compared (as described in Section 2.9) to determine the most suitable magnetic beads, as well as whether there was any significant difference in the use of DTT and TCEP. Lastly, DNA profiling was done on the fractions obtained from the top-performing magnetic bead combination (Section 2.7 and Section 2.8).

#### 2.5.2. Phase 2: Optimisation of the Initial DDE Method

The DDE method selected in Phase 1 included the use of 400 nm SeraSil-Mag^TM^ for separation, NucleoMag^®^ F-beads for purification of the sperm fraction and DTT for sperm lysis. Optimisation of this initial DDE method was then performed to (i) minimise premature sperm cell lysis within the initial epithelial cell lysis incubation step, and (ii) ensure the sample-to-beads ratio was appropriate for adequate removal of epithelial cells during the separation step.

**Figure 3:**
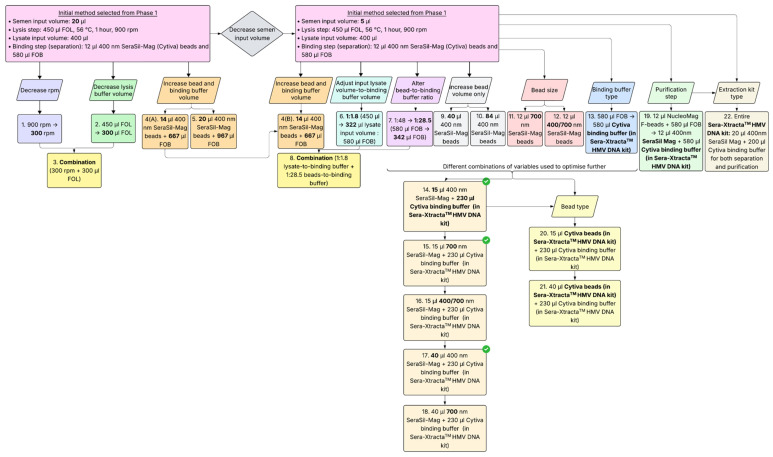
Summary of the method adjustments (1–22) made to the epithelial cell lysis and epithelial cell binding steps to improve epithelial and sperm DNA separation. The method adjustments indicated by the green tick (14, 15 and 17) were identified as the three top-performing and were selected for evaluation in Phase 3 (created using Lucidchart. Publicly available at https://lucid.co/lucidchart; date accessed 14 July 2026).

The method adjustments made are provided here (Figure 3), but a detailed description of each change is included in the Supplementary Materials (Table S1).

Variables adjusted during initial optimisation of the epithelial cell lysis incubation and epithelial cell binding steps were (1) the revolutions per minute (rpm), which saw the shaking speed decrease from 900 rpm to 300 rpm, (2) the lysis buffer (FOL) volume, which was reduced from 450 μL to 300 μL, (3) decreased lysis shaking (300 rpm) with reduced (300 μL) lysis buffer (FOL) volume, and lastly the magnetic bead volume was increased from 12 μL to (4(A)) 14 μL, and (5) to 20 μL (with the binding buffer maintaining the original ratio of 1:48).

After the semen volume was reduced to 5 μL, the increased magnetic bead volume (14 μL) was re-evaluated (4(B)). Additionally, method adjustments 6–22 were systematically performed, and the assessed variables included: (6) the lysate volume-to-binding buffer ratio (adjusted from 1:1.45 to 1:1.8), as well as (7) the bead-to-binding buffer ratio (adjusted from 1:48 to 1:28.5 [35]) and (8) a combination of the adjusted input lysate-to-binding buffer ratio (1:1.8) and bead-to-binding buffer ratio (1:28.5). The magnetic bead volume was increased from 12 μL to (9) 40 μL and (10) 84 μL, without adjustment to the binding buffer. The bead size used for separation was also changed from 400 nm to (11) 700 nm and (12) a 50:50 mixture of 400 nm and 700 nm. The separation step binding buffer type was changed from binding buffer FOB (NucleoMag^®^ Forensic DNA kit) to (13) the binding buffer included in the Sera-Xtracta^TM^ HMV DNA kit (Cytiva, Marlborough, MA, USA).

Method adjustments 14–18 were then evaluated, and different combinations of these previously listed variables, specifically focusing on the bead-to-binding buffer ratio outlined in the Sera-Xtracta^TM^ HMV DNA kit handbook [36], bead size and binding buffer type: (14) 15 μL 400 nm beads and 230 μL Cytiva binding buffer; (15) 15 μL 700 nm beads and 230 μL Cytiva binding buffer; (16) 15 μL 400/700 nm beads (50:50 mixture) and 230 μL Cytiva binding buffer; (17) 40 μL 400 nm beads and 230 μL Cytiva binding buffer, and (18) 40 μL 700 nm beads and 230 μL Cytiva binding buffer.

Thereafter, the magnetic beads and binding buffer used in the purification step were changed from NucleoMag-F^®^ beads and binding buffer FOB (from the NucleoMag^®^ DNA Forensic kit) to (19) 400 nm SeraSil-Mag^TM^ beads and the Sera-Xtracta^TM^ HMV DNA kit binding buffer. Method adjustments 20–21 focused on changing the bead type used for separation from the SeraSil-Mag^TM^ to (20) 15 μL of the Cytiva beads within the Sera-Xtracta^TM^ HMV DNA kit and (21) 40 μL of the Cytiva beads within the Sera-Xtracta^TM^ HMV DNA kit. Lastly, the extraction kit itself was assessed (22), and the entire protocol was performed using the 400 nm SeraSil-Mag^TM^, coupled with the Sera-Xtracta HMV DNA kit.

A DNA extraction blank was included in each experiment, and all DNA was quantified (Section 2.6) and profiled (Section 2.7 and Section 2.8). Data analysis was performed according to Section 2.9 to evaluate the sperm fraction Y-target DNA yield, M:F ratio, presence of male alleles, and log(LR) for each method adjustment prior to selection in Phase 3.

#### 2.5.3. Phase 3: Further Evaluation of the Top Three Performing Methods

In Phase 2, method adjustments 14, 15, and 17 were identified as the top-performing and were therefore applied again in Phase 3, but to reduced biological material. These method adjustments utilised binding buffer from the Sera-Xtracta^TM^ HMV DNA kit (230 μL) in combination with: (14) 15 μL of 400 nm SeraSil-Mag^TM^ beads, (15) 15 μL of 700 nm SeraSil-Mag^TM^ beads, and (17) 40 μL of 400 nm SeraSil-Mag^TM^ beads for separation, respectively.

The resultant fractions were quantified (Section 2.6) and profiled (Section 2.7 and Section 2.8). Again, the sperm fraction Y-target DNA yield, M:F ratio, male allele presence, and log(LR) were evaluated (Section 2.9) to identify a final method for authentic post-coital swab processing (Supplementary Materials Figure S1).

### 2.6. DNA Quantification

DNA was quantified using the Quantifiler^TM^ Trio DNA Quantification Kit (Applied Biosystems, Foster City, CA, USA), according to the manufacturer’s protocol [37], except that half-volume reactions were used. This kit was used as it includes a small autosomal target (80 base pairs (bp)), a large autosomal target (214 bp), and a male-specific Y-target (75 bp) [37]. The 7500 Real-Time PCR System (Applied Biosystems, Foster City, CA, USA) was used, and a no-template control (NTC) was included in each experiment.

The quantification data were analysed on the HID Real-Time PCR Analysis software v1.2 (Applied Biosystems, Foster City, CA, USA). In particular, the Y-target yield and M:F ratio of the resultant sperm fractions per experiment were evaluated. The M:F ratio is based on the concentration of the male-specific Y-target in relation to the small autosomal target (minus the Y-target concentration) [37].

For context, the South African Police Service (SAPS) Forensic Science Laboratory requires the sperm fraction to have a Y-target concentration of at least 0.004 ng/μL (which equates to 0.12 ng in this study, where DNA yield was reported as opposed to concentration) and a M:F ratio not exceeding 1:10 to proceed to DNA profiling (personal communication, 2025). These “thresholds” were used as a point of comparison for this study.

### 2.7. DNA Profiling

DNA profiling was performed using the Globalfiler^TM^ PCR Amplification Kit (Applied Biosystems, Foster City, CA, USA) according to the manufacturer’s protocol [38], except that half-volume reactions were used. DNA was normalised to 0.5 ng based on the average autosomal target concentration (i.e., the small and large autosomal targets), using molecular biology grade (MBG) water, if required.

DNA amplification was performed using the T100 thermal cycler (BioRad Laboratories, Hercules, CA, USA), and the resultant PCR products were separated using the 3500xL Genetic Analyser (Applied Biosystems, Foster City, CA, USA) with a 36 cm capillary array and POP4 polymer. The following capillary electrophoresis (CE) conditions were used: 1.2 kV injection voltage, 15 s injection time, 13 kV run voltage and 1550 s run time. An NTC was included in each experiment.

### 2.8. DNA Profile Interpretation

The TrueAllele^®^ system (Cybergenetics, Pittsburgh, PA, USA) was explored to provide accurate quantitative modelling and LR measurements for DNA mixture interpretation in an automated manner. No thresholds were applied to the quantitative STR peak data. The resultant log(LR) values were used to quantify the relative efficacy of different DNA extraction methods. The software also computed additional genotype statistics, such as DNA match accuracy [39] and LR error rates [40].

DNA profiles were interpreted using TrueAllele^®^ server version 3.25.8088.1, and reviewed in VUIer^TM^ client software version 3.3.8343.1R20b (Cybergenetics, Pittsburgh, PA, USA). For each sperm fraction sample, 100,000 MCMC iterations were used, and two log(LR) values were generated, one where the female contributor to the original mixture remained unknown (non-peeling), and another where the female was known and stipulated as a prior condition (peeling). Each sample was run twice, in two independent computer runs.

For comparative purposes, GeneMapper^®^ *ID-X* Software Version 1.5 (Thermo Fisher Scientific, Waltham, MA, USA) was used to view resultant DNA profiles, with an analytical threshold (AT) of 50 relative fluorescence units (RFU) and a stochastic threshold (ST) of 200 RFU. DNA profiles obtained from the resultant sperm fractions with all male alleles present at each marker were noted, as this is typically a requirement at SAPS (personal communication, 2025). However, this was not used as a metric for method evaluation, as resultant sperm fraction samples underwent probabilistic genotyping, regardless of male allele presence.

### 2.9. Statistical Analysis

Descriptive statistics were calculated using Microsoft^®^ Excel^®^ (Version 2604) (Redmond, WA, USA). Significance testing was performed using IBM SPSS Statistics (version 31.0.0.0 (117)) (Armonk, NY, USA). A Shapiro–Wilk test for normality was performed. For independent samples, a one-way ANOVA with Tukey’s HSD test was used to perform multiple pairwise comparisons if data were normally distributed, and if data were not normally distributed, a Mann–Whitney U test was performed to compare two groups, while a Kruskal–Wallis test was performed to compare more than two groups. In Phase 1, outliers were removed prior to significance testing of the expanded sample size to avoid undue inflation of the mean Y-target yield. For related samples, a parametric paired *t*-test or non-parametric Wilcoxon signed rank test was used where appropriate. A Bonferroni correction was applied for multiple testing. Spearman’s rho test was used to assess correlation.

## 3. Results

Full datasets for Phases 1, 2 and 3 are available in the Supplementary Materials (Tables S2, S3 and S5, respectively).

### 3.1. Phase 1: Establishment of the Initial DDE Method Outcomes

**Figure 4:**
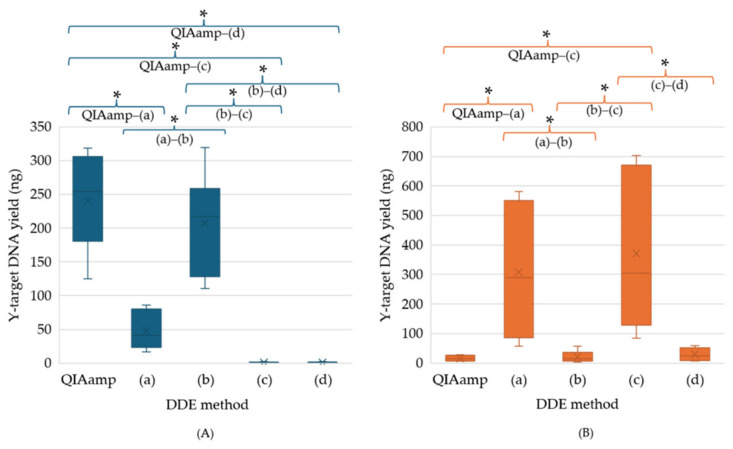
The Y-target DNA yield obtained for the initial DDE method investigation (*n* = 8 samples per combination): (**A**) the sperm fraction (significance tested with ANOVA); (**B**) the epithelial fraction (significance tested with Kruskal–Wallis). Significant differences are indicated by asterisks (*) (*p* < 0.005). The mean per box-and-whisker plot is indicated by “X”. The baseline method is indicated by “QIAamp”. Magnetic bead combinations: (a) NucleoMag^®^ F-beads + NucleoMag^®^ F-beads; (b) 400 nm SeraSil-Mag^TM^ + NucleoMag^®^ F-beads; (c) NucleoMag^®^ F-beads + NucleoMag^®^ NGS Clean-up and Size Select; (d) 400 nm SeraSil-Mag^TM^ + NucleoMag^®^ NGS Clean-up and Size Select.

Phase 1 focused on establishing the initial DDE method using the NucleoMag^®^ Forensic DNA kit by adding differential lysis steps to the manufacturer’s relatively generic protocol for “Forensic samples”. Different magnetic beads were evaluated for the separation and purification steps in a total of four distinct bead combinations (a–d) (Figure 2). The resultant DNA was quantified, which showed significantly higher Y-target DNA yields in the sperm fraction when magnetic bead combination (b) was used (mean = 207 ng (standard deviation (SD) = 75.08 ng)). This was followed by combination (a) (mean = 47.69 ng (SD = 28.51 ng)) (Figure 4A). Both of these combinations utilised NucleoMag^®^ F-beads for purification; thus, these magnetic beads were selected for purification of the sperm fraction for all further experimentation. Combinations (a) and (b) each used different magnetic beads for separation, which warranted further analysis.

For the epithelial fraction, significantly lower Y-target DNA was obtained when combination (b) (median = 15.05 ng, range = 4.02 ng–57.38 ng) or (d) (median = 25.39, range = 7.62 ng–59.04 ng) was used compared to combinations (a) and (c). This indicated that little male DNA was carried over into the epithelial fraction when combinations (b) and (d) were used. These combinations both used 400 nm SeraSil-Mag^TM^ for separation (Figure 4B).

Furthermore, the investigation showed that when bead combination (b) was used, the Y-target DNA yield in both the sperm and epithelial fractions was comparable to that obtained from our baseline method—the widely used QIAamp^®^ DNA Investigator Kit (Figure 4).

The DDE method involving magnetic bead combination (b) was then applied to an additional expanded sample size (*n* = 5). Using a Mann–Whitney U test, no significant difference was observed in the Y-target DNA yield between the original and additional samples (*p* = 0.808; α = 0.05), thus confirming the reliability of the results.

**Figure 5:**
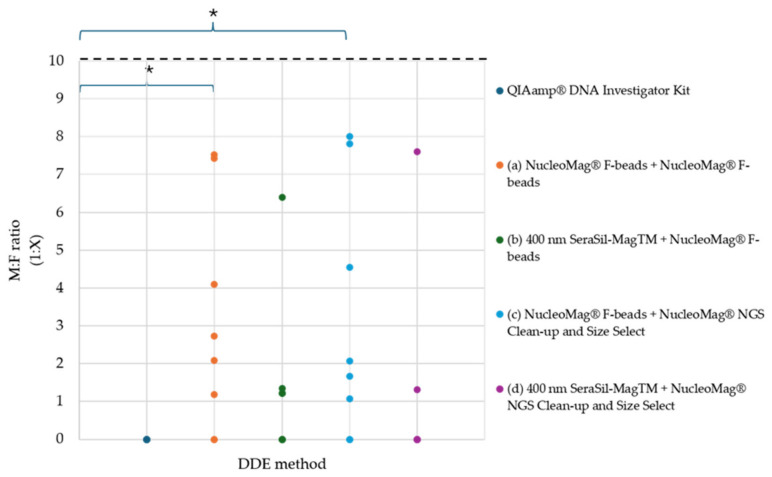
The M:F ratios obtained for the sperm fraction of each DDE method investigated (*n* = 8 samples per combination). A Kruskal–Wallis test was used to determine significance, indicated by an asterisk (*) (*p* < 0.005). The dotted line indicates the 1:10 “threshold”.

The M:F ratio for the sperm fractions was also considered. Figure 5 shows that the sperm fraction M:F ratio for samples extracted using the baseline method is represented by a value of “0”, indicating no or negligible female DNA carryover into any of the sperm fractions. Comparison of the different magnetic bead combinations (a–d) revealed that the DDE methods (b) and (d) performed the closest to the baseline method (b): median = 1:0.61, range = 0–1:6.40; (d): median = 0, range = 0–1:7.60) (Figure 5).

DNA profiling was carried out on all DNA samples where magnetic bead combination (b) was used. GeneMapper^®^ *ID-X* Software Version 1.5 revealed that full DNA profiles for the sperm fraction were obtained from mock sexual offence samples processed using the DDE method (b) for all but two samples. Figure S2 (Supplementary Materials) shows two channels of a DNA profile, where the full presence of the male alleles within a resultant sperm fraction can be observed.

The sperm fractions were analysed using the TrueAllele^®^ system, and the correct male contributor was identified for each sample with statistical confidence (non-peeling: median = 20.56, range = 13.29–30.6); peeling: median = 29.19, range = 19.51–30.67 (values based on the average of the two log(LR) replicates per sample)) (Table S1).

Lastly, the efficiency of two sperm cell-reducing agents was also investigated in Phase 1. A Mann–Whitney U test revealed no significant difference between the use of DTT (median = 12.84 ng, range = 0.25 ng–260.37 ng) and TCEP (median = 9.17 ng; range = 0.17 ng–319.81 ng) (*p* = 0.867; α = 0.05). Due to availability, DTT was selected for future use.

### 3.2. Phase 2: Optimisation of the Initial DDE Method Outcomes

#### 3.2.1. Metric-Based Assessment

**Figure 6:**
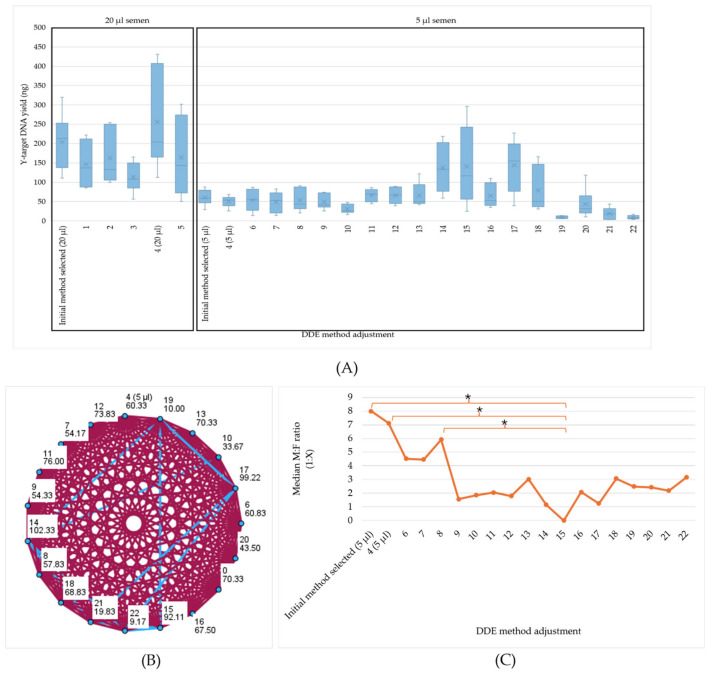
(**A**) Overview of the Y-target DNA yield (ng) obtained from mock sexual offence samples processed using different DDE method adjustments in Phase 2 (*n* = 6 samples per method adjustment; top three method adjustments: 3 additional samples). (**B**) Pairwise comparisons of the Y-target DNA yield (ng) of the DDE method adjustments for 5 μL semen obtained using the Kruskal–Wallis test. “0” indicates the initial selected method (5 μL). Significant differences are indicated in blue (*p* < 0.0003). Each node shows the sample average rank of the DDE method (generated by IBM SPSS Statistics (version 31.0.0.0 (117))). (**C**) Median of the M:F ratio obtained for the sperm fraction of mock sexual offence samples processed per DDE method adjustment with a 5 μL semen input volume. Significant differences indicated by the asterisks (*) (*p* < 0.0003).

Phase 2 focused on optimising the DDE method established in Phase 1 using magnetic bead combination (b) by altering different variables within the method and systematically evaluating DNA quality metrics and DNA profile log(LR)s. Basic method adjustments (1–5) were made to the initial method to improve separation. ANOVA revealed no significant differences between the Y-target DNA yield of these method adjustments (*p* = 0.052; α = 0.003) (Figure 6A); thus, the initial method was re-established using mock sexual offence samples prepared with reduced semen volume (5 μL) and further optimisation was compared to this “new” initial method (method adjustments 4 (B), 6–22).

A predicted decrease in Y-target DNA yield was observed when semen volume was decreased to 5 μL. Comparison within the 5 μL semen volume group showed that method adjustments 14, 15 and 17 had the highest median Y-target DNA yield (14: median = 133.35 ng, range = 58.91 ng–218.79 ng; 15: 116.24 ng, range = 25.20 ng–296.07 ng; 17: median = 155.26 ng, range = 39.48 ng–227.06 ng) (Figure 6A). A Kruskal–Wallis test revealed that method adjustments 14, 15 and 17 significantly differed from method adjustments 19, 21 and 22, respectively (Figure 6B). This provided support for these three method adjustments performing most optimally.

Evaluation of the M:F ratios obtained within the 5 μL semen volume group displayed a similar trend, whereby method adjustments 14, 15 and 17 had the lowest median M:F ratio in relation to the rest of the method adjustments (14: median = 1:1.15, range = 0–1:8.08; 15: median = 0, range = 0–1:2.14; 17: median = 1:1.25, range = 0–1:4.16), suggesting little female DNA carryover into these sperm fractions (Figure 6C).

A review of the Amelogenin Y-chromosome peak heights (RFU) across the sperm fraction DNA profiles of all the method adjustments revealed that method adjustments 14, 15 and 17 produced the top three medians (14: 3898 RFU, 15: 5214 RFU, 17: 4115 RFU) (Table S4). The male autosomal allele presence within these method adjustments was averaged at 99.47%, 100% and 98.41%, respectively.

Information-preserving genotyping was performed using non-peeling conditions on the sperm fraction samples for the initially selected methods, as well as each method adjustment. Interestingly, for the method adjustments focusing on 5 μL semen, the highest median log(LR) value was obtained by method adjustment 19 (median = 23.35, range = 18.46–25.68), while method adjustments 14, 15 and 17 obtained median log(LR) values of 21.59 (range = 10.31–28.12), 18.13 (range = 5.6–30.99) and 13.25 (range = 1.91–23.80), respectively (values based on the average of the two log(LR) replicates per sample) (Tables S3 and S4). However, no significant differences were observed between the log(LR) values obtained for any of the method adjustments evaluated (*p* > 0.00017). Furthermore, the TrueAllele^®^ system was able to consistently identify the correct male contributor within the resultant sperm fractions, where sufficient DNA information was available.

These combined results showed that method adjustments 14, 15 and 17 outperformed the other method adjustments evaluated. Overall, no significant differences between these three method adjustments were observed, but further insight into their performance was expected from evaluation on mock sexual offence samples containing less biological material. These method adjustments were therefore applied in Phase 3 when even less biological material was used to create mock sexual offence samples.

#### 3.2.2. Metric Correlation Investigation

Correlations were investigated to determine whether the quantitative metrics, such as Y-target DNA yield (ng) and M:F ratio, or male allele presence within a DNA profile (Amelogenin Y-peak height (RFU)), could be used to inform log(LR) values obtained from the TrueAllele^®^ system. Spearman’s rho test showed that weak positive correlations existed between the Y-target DNA yield and Amelogenin Y peak height (ρ = 0.279), as well as log(LR) values (ρ = 0.307). A weak positive correlation was also identified between the Amelogenin Y peak height and log(LR) values (ρ = 0.388). Moderate negative correlations were identified between the M:F ratio and Y-target DNA yield (ρ = −0.533) and log(LR) values (ρ = −0.440), while a strong negative correlation was found between the M:F ratio and Amelogenin Y peak height (ρ = −0.627) (Table 1).

### 3.3. Phase 3: Further Evaluation of the Top Three Performing Methods Outcomes

#### 3.3.1. Comparison of Input Biological Material

**Figure 7:**
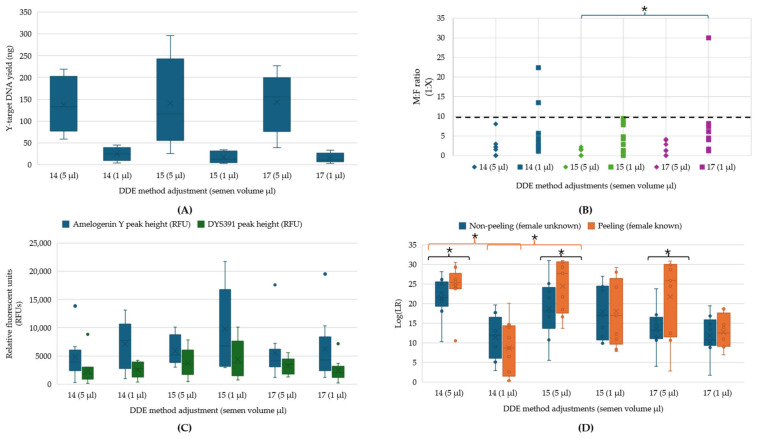
A comparison of the results obtained for mock sexual offences processed in Phase 2 (5 μL semen) and Phase 3 (1 μL semen) using the top three performing method adjustments (14, 15 and 17) selected from Phase 2 (*n* = 9 samples per method adjustment). Significance is indicated by the asterisks (*) (*p* < 0.0033). (**A**) Y-target DNA yield (ng). (**B**) M:F ratios, with the “threshold” indicated by the dotted line (1:10). (**C**) the Amelogenin Y peak and DYS391 peak heights in RFU. (**D**) the male contributor log(LR) values (>0) using non-peeling and peeling conditions.

An ANOVA assessment of the Y-target DNA yield (ng) between the method adjustments revealed an expected significant decrease between mock sexual offence samples prepared with 5 μL and 1 μL of semen (*p* < 0.0033). However, although method adjustment 14 had the highest mean Y-target DNA yield (mean = 25.04 ng (SD = 15.11 ng)), no significant difference was observed between method adjustments 14, 15 and 17 for mock sexual offence samples prepared with 1 μL of semen (Figure 7A).

Evaluation of the M:F ratios for mock sexual offence samples prepared with 1 μL of semen showed majority of the sperm fraction samples had a M:F ratio below 1:10, with the exception of two outliers identified in method adjustment 14 and a single outlier identified in method adjustment 17. Method adjustment 14 had the lowest median M: F ratio (1:3.53, range = 1:1.08–1:22.46), while method adjustment 15 showed the least variation (median = 1:4.20, range = 0–1:9.49). Comparison between the 5 μL and 1 μL semen input volume groups showed that the only significant difference observed was between method adjustment 15 (5 μL) and method adjustment 17 (1 μL) (Figure 7B).

Male allele presence within the resultant sperm fraction DNA profiles for mock sexual offence samples prepared with 1 μL of semen was assessed. The percentage of present autosomal alleles was 98.94%, 99.47% and 99.21% for method adjustments 14, 15 and 17, respectively. These results are similar to those previously described for the 5 μL semen input volume group. Further comparison between mock sexual offence samples prepared with 5 μL semen and 1 μL semen was conducted, whereby the heights of the Amelogenin Y peak and the male-specific marker (DYS391) were considered. A Kruskal–Wallis test showed no significant differences between these metrics (Figure 7C). The highest Amelogenin Y peak (21,758 RFU) and DYS391 peak (10,110 RFU) were identified in method adjustment 15 (1 μL).

Match statistics were also considered for method adjustments 14, 15 and 17. The informativeness of two prior conditions, namely non-peeling and peeling, was investigated, and results revealed that within the 5 μL semen input volume group, the peeling log(LR) values for the male contributor were significantly greater than the non-peeling log(LR) values for method adjustments 14, 15 and 17, while in the 1 μL semen input volume group, no significant differences were observed (Figure 7D). It was noted, however, that even with the peeling prior condition applied, the 1 μL semen input volume group had some sperm fraction samples showing greater log(LR) values for the female contributor (Table S3). This was not the case for the 5 μL semen input volume group.

When considering the log(LR) values obtained for the male contributor across the method adjustments, ANOVA revealed no significant difference between the non-peeling results for method adjustments 14, 15 and 17 for mock sexual offence samples prepared with 5 μL and 1 μL semen. A significant difference was noted for peeling results between method adjustments 14 (5 μL) and 14 (1 μL), as well as 14 (1 μL) and 15 (5 μL).

Lastly, sperm fraction samples, which would typically not progress within the forensic DNA profiling workflow due to failure to meet binary thresholds, were identified for method adjustments 14, 15 and 17 across the two semen input volume groups (Table 2). Although all seven samples met the DNA yield threshold, several samples (42.86% (*n* = 3/7)) failed to meet the M:F ratio threshold. Furthermore, when considering the DNA profiles, the data for all seven samples would not have been reported on due to the missing male autosomal alleles identified during GeneMapper^TM^ visualisation. Despite this, positive log(LR) values were obtained for these samples.

Overall, these results indicated that although the mock sexual offence samples prepared in Phase 3 had a lower semen volume input, information-preserving genotyping was able to resolve the mixture into a male and female contributor with statistical confidence, regardless of semen input volume.

#### 3.3.2. Comparison to an Authentic Post-Coital Swab

The combined results from the mock sexual offence samples processed in Phases 2 and 3 showed negligible significant difference between method adjustments 14, 15 and 17, and therefore method adjustment 14 was selected to be applied to an authentic post-coital swab. The sperm fraction obtained from the post-coital swab revealed that the Y-target DNA yield achieved was similar to that of the mock sexual offence sample prepared with 1 μL semen and a five-stroke buccal swab; however, the M:F ratio indicated high female DNA carryover (Table 3). This was echoed in the match statistics, which revealed that only the female contributor could be identified by the TrueAllele^®^ system for both non-peeling and peeling conditions.

## 4. Discussion

Sexual offence samples present as a major challenge for many forensic laboratories due to the lengthy and/or inadequate DNA analysis and interpretation commonly utilised. These challenges often hinder justice and may, in fact, be avoidable through the use of modern workflows, which prioritise information preservation [39]. In this context, this study aimed to demonstrate proof-of-concept that magnetic beads can be used to separate epithelial DNA from the sperm fraction in sexual offence samples. This innovation theoretically enables the entirety of the DDE method to be automated for high-throughput processing, as it eliminates the need for centrifugation. We also explored how DNA interpretation using the TrueAllele^®^ system can preserve information of resultant DNA profiles and compared this approach to applying binary thresholds that are commonly adopted in forensic casework laboratories.

In Phase 1 of this study, a DDE method using magnetic beads for the separation of epithelial and sperm fractions was successfully established (Figure 2). This method utilised the NucleoMag^®^ Forensic DNA kit, whereby the current protocol was adapted to create a novel DDE protocol. This kit was selected as it is compatible with automation and complies with ISO 18385 requirements [34]. Furthermore, established semi-automated DDE methods already exist for other well-known manufacturers [12,13,14], highlighting this as an under-explored kit.

This kit was coupled with the use of 400 nm SeraSil-Mag^TM^ for separation and NucleoMag^®^ F-beads for purification. The selection of this magnetic bead combination for use within the DDE method was motivated by the quantification results, which showed this combination significantly outperformed the other combinations investigated by achieving the highest mean and median sperm fraction Y-target DNA yield and the lowest mean and median epithelial fraction Y-target DNA yield (Figure 4). This suggested that female DNA was sufficiently removed during the mixture separation step, retaining the male DNA within the sperm fraction. This was further supported by the M:F ratios obtained for this magnetic bead combination (Figure 5). A median M:F ratio was determined as 1:0.61, indicating little female DNA carryover into the sperm fraction and that separation of the mixture into a male and female component did in fact occur.

The magnetic beads used in this study differ from those previously described in the literature. If used for separation, magnetic beads have been conjugated with sperm-specific antibodies to achieve separation of sperm and other cells [30,41,42]. The MACSprep™ Forensic Sperm MicroBead Kit [27] is a commercially available example; however, the protocol still requires centrifugation, thus limiting its automatability and sample throughput.

While both bead types utilised in this study are superparamagnetic, the SeraSil-Mag^TM^ beads are silica-coated [43] and the NucleoMag^®^ F-beads consist of carboxylated particles. Both bead types are used for nucleic acid isolation from samples with small amounts of DNA; however, the NucleoMag^®^ F-beads are specifically designed for forensic casework samples [34].

In Phase 2, the selected DDE method was optimised in a systematic manner (Figure 3). Although a total of 24 method adjustments were made, only those applied to more representative mock sexual offence samples (4 (B), 6–22) were considered for further evaluation. A comparative review of the results obtained per method adjustment (Figure 6, Table S4) revealed that the key adjustment made was to the buffer type variable, whereby binding buffer FOB was replaced with the Sera-Xtracta^TM^ HMV DNA kit binding buffer for the separation step, specifically.

Three method adjustments, namely 14, 15 and 17, utilising SeraSil-Mag^TM^ beads and Sera-Xtracta^TM^ HMV DNA kit binding buffer for separation, differing only in bead size and/or bead volume, were distinguished from the rest based on their superior quantification performance (Figure 6). Furthermore, full male DNA profiles were obtained for 25 of the 27 resultant sperm fraction samples assessed across these three method adjustments, and interpretation of these DNA profiles returned informative log(LR) values (Tables S3 and S4). Importantly, no significant differences were observed between these three method adjustments. It was therefore hypothesised that these improved results were attributed to improved chemistry between the magnetic bead and binding buffer, allowing for increased binding capacity for epithelial DNA during separation. The magnetic bead binding capabilities are largely affected by the environment in which they are used. Binding buffers are essential to this environment and are designed to provide the appropriate amount of chaotropic salts and/or pH, at a certain bead-to-buffer ratio, required for the coated particles within the magnetic bead solution to function optimally [44]. Interestingly, the SeraSil-Mag beads (400 nm and 700 nm) do not form part of the Sera-Xtracta^TM^ HMV DNA kit. Therefore, the same hypothesis was applied to evaluate the beads supplied directly within the Sera-Xtracta^TM^ HMV DNA kit at different volumes, along with the Sera-Xtracta^TM^ HMV DNA kit binding buffer (method adjustments 20 and 21); however, these performed less effectively than method adjustments 14, 15 and 17.

In Phase 3, the three method adjustments were applied to mock sexual offence samples that were prepared with reduced input of epithelial cells and sperm cells. Comparison of these data to Phase 2 showed that the only significant difference observed between quantification metrics obtained from the DNA extracted from the two different input amounts was the sperm fraction Y-target DNA yield (Figure 7A), while other metrics evaluated for the sperm fraction, including M:F ratios (Figure 7B), DNA profile Y-specific peak heights (Figure 7C), and male allele presence remained relatively similar. This suggested that the separation and purification capabilities of method adjustments 14, 15 and 17 remained consistent across different semen input volumes.

Direct comparison of these quantitative metrics to other available literature is limited due to the inconsistencies observed, particularly in mock sexual offence sample preparation across studies, in terms of female sample type and male DNA input, as well as differences in the DDE method investigated, and chemistries of the DNA quantification and DNA profiling kits utilised. Nonetheless, assessment of the quantification M:F ratios obtained in a study investigating a modified differential lysis method [45] revealed comparative results to those obtained within this study (Tables S2, S3 and S5). Hudson & Green (2024) performed DNA quantification using the Investigator^®^ Quantiplex HYres Kit (QIAGEN, Hilden, Germany) and obtained sperm fraction M:F ratios of between 1:1.40 and 1:29.80 [45]. Furthermore, our study displayed larger male allele peak heights in the sperm fraction DNA profiles, even for mock sexual offence samples prepared with the lowest semen input volume investigated (1 μL). However, it is important to note that normalisation of the sperm fraction samples for profiling according to the average autosomal concentration typically reduced the presence of the male DNA within the sample, especially in Phase 3, thereby complicating interpretation.

To overcome the complexity of manual DNA mixture interpretation, automated interpretation was investigated for all method adjustments in Phases 2 and 3. Similar to manual DNA mixture interpretation, probabilistic genotyping outcomes are also affected by the quality of the DNA profile; however, the TrueAllele^®^ system consistently resolved the mixed sperm fraction samples into the correct male and female contributors, with relatively high statistical confidence as indicated by the returned log(LR) values (Tables S3 and S5).

A correlation analysis investigating the relationship between the qPCR metrics (Y-target DNA yield and M:F ratio), Amelogenin Y peak height within the DNA profile, and match statistics (log(LR) values) was conducted using the resultant sperm fractions from the Phase 2 method adjustments (Table 1). The log(LR) values are the final output within this study’s workflow; therefore, the use of the other metrics to inform this warranted exploration. The greatest correlation informing the log(LR) values was observed as a moderate negative correlation with the M:F ratio (*ρ* = −0.440). This is expected as statistical support for the male contributor will increase as the female DNA carryover within the sperm fraction is reduced.

The results from this study suggest that automated interpretation through information-preserving genotyping allowed for mixture resolution despite some of the sperm fraction samples having M:F ratios exceeding 1:10 (indicated by the outliers identified in Figure 7B) and/or lower peak heights, or allele drop-out, within the DNA profiles (Table 2). For example, in Phase 3, a sperm fraction DNA profile originating from reduced biological input had a M:F ratio of 1:29.98 and a returned log(LR) value of 1.78 (non-peeling). These results highlight the potential systematic loss of DNA evidence using current DNA protocols, and are particularly relevant to the ongoing discussion around the use of quantification, interpretation and reporting thresholds [39,46], which in some laboratories determines the stage at which a DNA sample is processed up until within the forensic DNA workflow. The unnecessary removal of probative evidence from the forensic DNA workflow is a policy issue. Protocols which exclude sexual offence evidence based on binary thresholds may have been in place because the interpretation thereof is complex, and a modern solution was not yet available. However, this leaves victims and/or survivors without justice. With the increased availability of new probabilistic genotyping systems, policies and protocols should be updated accordingly.

The use of genotype conditioning in the TrueAllele^®^ system was also investigated, whereby the female contributor within a mixture is already known (i.e., peeling). This is typically the case when a female reference sample is collected and provides the TrueAllele^®^ system with more information to complete the analysis, which can be particularly useful when biological material from the male is limited (as in Phase 3). Significant increases were observed in log(LR) values for peeling conditions versus non-peeling conditions for samples processed in Phase 2 using method adjustments 14 and 15 and Phase 3 using method adjustment 14 (Figure 7D).

These data support the use of information-preserving genotyping using the threshold-free TrueAllele^®^ system, especially in complex interpretation. They once again highlight that the data thresholds implemented within the forensic workflow should be reconsidered. Furthermore, these results illustrate that complete separation of male and female DNA by a DDE method is not necessary, provided it is sufficient for reliable mixture resolution using the TrueAllele^®^ system.

Lastly, method adjustment 14 was selected as the final optimised buccal swab mock sexual offence DDE method and applied to an authentic post-coital swab. The results suggested that although adequate Y-target DNA yield was obtained, there was extreme female carryover into the sperm fraction (Table 1). This may perhaps be due to the differences noted between buccal and vaginal swabs (see Section 2.2). Although these results should be interpreted with caution, as only a single swab was investigated, this confirms the need for continued method optimisation using vaginal swab mock sexual offence samples and additional post-coital swabs. This further highlights the need for mock sexual offence sample standardisation for use within sexual offence method development. It is understood that using post-coital swabs for DDE method development studies is not feasible, as hundreds of samples from the same individuals would be required to systematically change one variable at a time. This is evident by the limited number of post-coital swabs included in similar studies [47,48], compared to the abundance of literature related to buccal swabs [27,28,29,30,31,32]. However, the classification of buccal swabs as a proxy is perhaps inaccurate, and this sample type should rather be considered as a rudimentary starting point for initial stages of development.

Therefore, although buccal swabs were considered appropriate to demonstrate proof-of-concept within this study, future research should focus on automation and evaluating this DDE method using more realistic mock sexual offence samples, such as those created with vaginal swabs and/or authentic post-coital swabs. Future studies should also compare this method’s performance with other available semi-automated methods, using samples prepared from the same set of donors, to place its performance into context with existing methods. Furthermore, it is important to acknowledge that, before this DDE protocol can be implemented operationally, rigorous validation experimentation must be conducted to assess the performance of the protocol under forensically relevant sexual offence casework scenarios.

## 5. Conclusions

This paper demonstrates the novel use of ordinary magnetic beads to separate mixed samples into sperm and epithelial fractions within a DDE method for the first time. It found that the 400 nm SeraSil-Mag^TM^ magnetic beads, coupled with the Sera-Xtracta^TM^ HMV DNA kit binding buffer, could achieve this. Additionally, this paper shows the advantages of using information-preserving mixture interpretation. In this study, threshold-free TrueAllele^®^ analysis could derive informative results even when conventional methods that discard data could not. Together, this DDE method and automated interpretation can theoretically contribute towards a fully automated DNA analysis workflow for sexual offence samples, with future work focusing on further optimisation and developmental validation. This may offer a solution to those countries, particularly in Africa, experiencing extreme sexual violence, and provide victims and/or survivors with justice.

## Figures and Tables

**Table 1:** Spearman’s rho correlation coefficient per metric relationship evaluated (*p* < 0.001). A negative relationship is indicated in blue, while a positive relationship is indicated in yellow.

	Y-Target DNA Yield (ng)	M:F Ratio (1:X)	Amelogenin Y Peak Height (RFU)	Log(LR) (Non-Peeling)
Y-target DNA yield (ng)	-	−0.533	0.279	0.307
M:F ratio (1:X)	-	-	−0.627	−0.440
Amelogenin Y peak height (RFU)	-	-	-	0.388
Log(LR) (non-peeling)	-	-	-	-

**Table 2:** Assessment of sperm fraction samples which do not meet the typically used binary thresholds (indicated by the bolded font) for the top three performing methods. The green indicates samples which produced eligible results. The red indicates the “stopping” point for these samples within the forensic DNA profiling workflow. The associated average of log(LR) values per sample, generated by the TrueAllele^®^ system, is also displayed. Graphics obtained from BioRender (https://www.biorender.com/).

			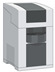		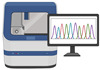	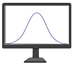	
Semen Input (μl)	Method Adjustment	Sample	Y-Target DNA Yield (ng)	M:F Ratio	Male Autosomal Allele Presence	Log(LR)	
						Non-Peeling	Peeling
			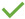 **0.12**	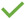 **≤ 1:10**	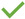 **No Missing Alleles**	**N/A**	**N/A**
5	14	SF_139	68.80	1:8.08	2 alleles missing	10.31	10.56
	17	SF_161	141.39	1:4.16	6 alleles missing	4.01	2.83
1	14	SF_204	7.34	1:22.46	3 alleles missing	-	0.21
		SF_207	3.54	1:13.49	1 allele missing	2.98	0.38
	15	SF_210	4.04	1:8.62	1 allele missing	9.78	7.81
		SF_213	3.05	1:9.49	1 allele missing	9.90	8.34
	17	SF_222	6.57	1:29.98	3 alleles missing	1.78	-

**Table 3:** Comparison of the metric results obtained for method adjustment 14 using three different sample types.

Sample Type	Y-Target DNA Yield (ng)	M:F Ratio	Non-Peeling Log(LR)	Peeling Log(LR)
Mock sexual offence Buccal swab (15 s), 5 μL semen	Mean = 137.54 (SD = 60.25)	Mean = 1:1.87 (SD = 2.60)	Mean = 21.54 (SD = 5.13)	Mean = 24.50 (SD = 5.62)
Mock sexual offence Buccal swab (5 strokes), 1 μL semen	Mean = 25.04 (SD = 15.11)	Mean = 1:6.36 (SD = 7.09)	Mean = 12.01 (SD = 5.41)	Mean = 9.46 (SD = 6.55)
Authentic post-coital	28.61	1:121.46	-	-

## Data Availability

The original contributions presented in this study are included in the article/Supplementary Material. Further inquiries can be directed to the corresponding author.

## Supplementary Materials

The following supporting information can be downloaded at https://www.mdpi.com/article/10.3390/genes17070824/s1: Table S1: Detailed description of the method adjustments made during the optimisation of the DDE method (Phase 2); Figure S1: A summary of the final DDE method; Table S2: A results summary for the differential DNA extraction methods evaluated in Phase 1; Figure S2: A DNA profile obtained using the bead combination selected in Phase 1; Table S3: A results summary for the differential DNA extraction method adjustments evaluated in Phase 2; Table S4: An overview of the metrics assessed for each method adjustment in Phase 2; Table S5: A results summary for the differential DNA extraction method adjustments applied in Phase 3.

## Abbreviations

The following abbreviations are used in this manuscript:

ANOVA	Analysis of variance
AT	Analytical threshold
bp	Basepair
CE	Capillary electrophoresis
cm	Centimetre
CPI	Combined probability of inclusion
DDE	Differential DNA extraction
DE	Differential extraction
DNA	Deoxyribonucleic acid
DTT	Dithiothreitol
HID	Human identification
HREC	Human Research Ethics Committee
HSD	Honestly significant difference
ISO	International Organisation of Standardisation
kV	Kilovolt
LR	Likelihood ratio
M:F	Male-to-female
MBG	Molecular grade water
MCMC	Markov Chain Monte Carlo
ng	Nanogram
NGS	Next-generation sequencing
nm	Nanometre
NTC	No-template control
PCR	Polymerase chain reaction
qPCR	Quantitative real-time PCR
RFU	Relative fluorescent unit
rpm	Revolutions per minute
SAPS	South African Police Services
SD	Standard deviation
ST	Stochastic threshold
STR	Short tandem repeat
TCEP	Tris(2-carboxyethyl)phosphine
UCT	University of Cape Town
UNICEF	United Nations Children’s Fund
USA	United States of America
μL	Microliter
