# Supplementary material for: A Novel Magnetic Bead-Based Differential DNA Extraction Method with Potential for High-Throughput Automation in Forensic Casework: A Proof-of-Concept Study
Source: Genes (Basel). 2026 Jul 19;17(7):824. doi: 10.3390/genes17070824 (PMC13409674; doi:10.3390/genes17070824)
Supplement: Supplementary file 1 [file genes-17-00824-s001.zip › Figure_S2.pdf]

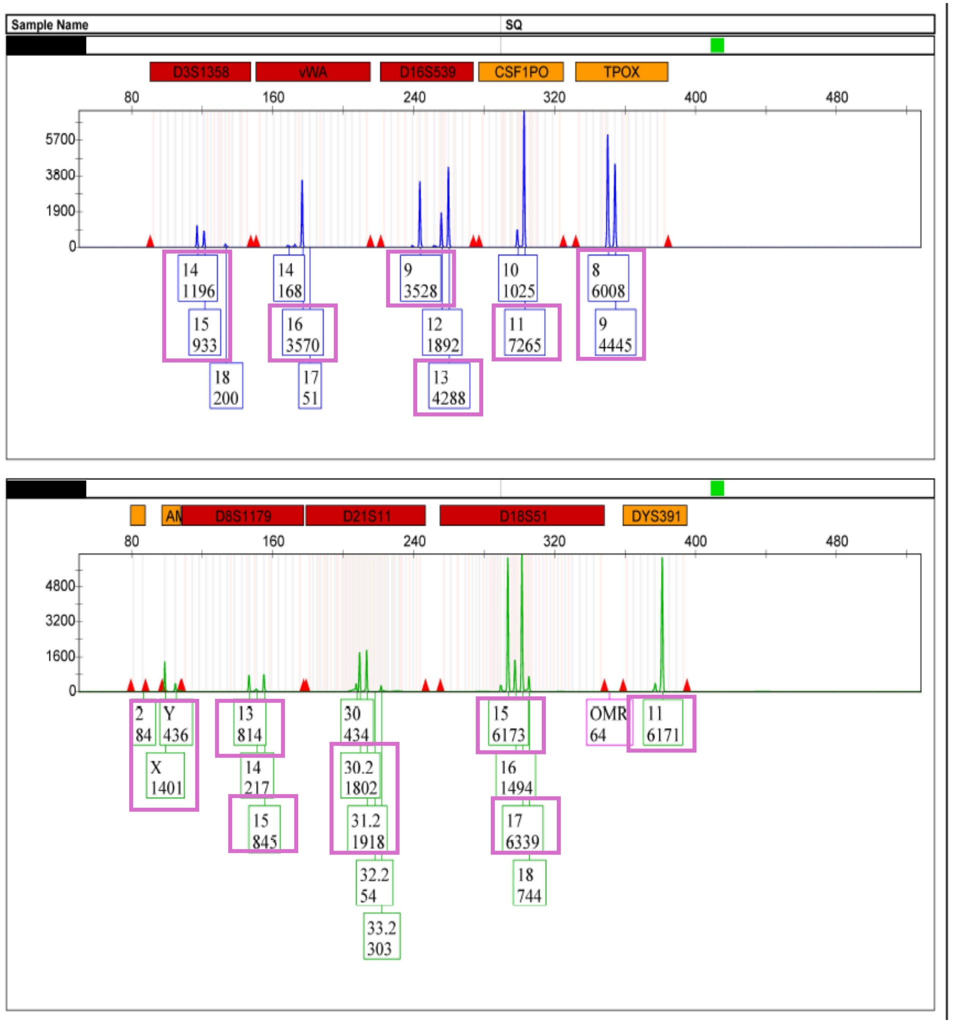

Figure S2. Blue and green channels from a sperm fraction DNA profile processed using DDE method (b). All male alleles were present at each marker in the DNA profile and are indicated by the purple squares.
