# Supplementary figures and images for: A Novel Magnetic Bead-Based Differential DNA Extraction Method with Potential for High-Throughput Automation in Forensic Casework: A Proof-of-Concept Study
Source: Genes (Basel). 2026 Jul 19;17(7):824. doi: 10.3390/genes17070824 (PMC13409674; doi:10.3390/genes17070824)

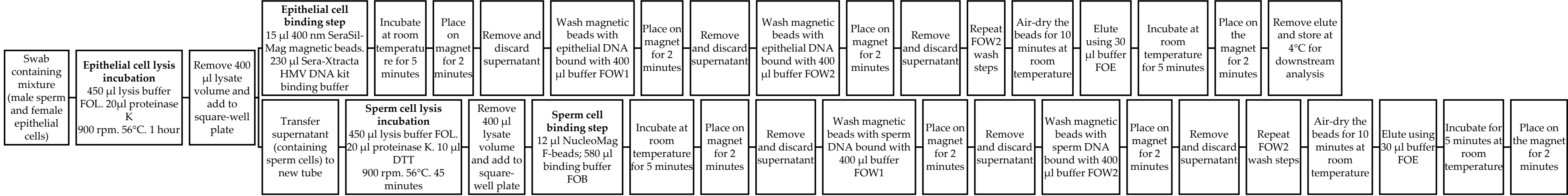

**Figure S1.** A summary of the final DDE method established within this study.

Supplement: Supplementary file 1 [file genes-17-00824-s001.zip › Figure_S1.pdf]
